# Adopting Clean Technologies to Climate Change Adaptation Strategies in Africa: a Systematic Literature Review

**DOI:** 10.1007/s00267-022-01704-w

**Published:** 2022-08-26

**Authors:** Ephraim Daka

**Affiliations:** Department of International Business, Turku University, Turku, Finland

**Keywords:** Air pollution, Environment, Wood fuel, Clean technology, Climate, Systematic literature review

## Abstract

The phenomenon of deforestation is occurring globally, in different types of forests, and for various reasons. In Africa, an estimated 90 per cent of the entire continent’s population uses wood as a source energy for heating and cooking. However, the unsustainable harvesting of trees for heating energy not only contribute to forest and environmental degradation, but it is also a significant contributor to ill health, air pollution and climate change. Reducing the resulting adverse of ecological and health consequences will have to involve a mix of adopting renewable fuels and natural resource sustainable strategies. To date, implementing this mix has shown significant challenges, especially in developing countries. We performed this systematic literature review (SLR) to help us better understand how research is approaching this mix in Africa. We screened 792 articles resulting in a final selection of 34 studies concerned with environmental sciences. The review drew on qualitative, quantitative, and regional studies and applying a standardized method for screening, data extraction and synthesis. The findings reveal that current research focuses dominantly in four key areas: (1) renewable technology transfer, (2) climate change-adaptability, (3) climate policy, and (4) technology adoption. However, we identified a literature gap on the thin literature concerned with the impact of clean technologies to improving the environment and people’s wellbeing. We propose planning a tool that would facilitate this process and suggest further research to incorporate monitoring its effectiveness.

## Introduction

Access to various forms of energy affects every aspect of economic development and livelihoods across the globe. During the decades, the demand for energy, especially for developing countries, has increased with a negative effect on the environment and livelihoods, which are critical sustainability challenges (Karanja and Gasparatos 2019). However, despite the growing development of renewable energies, developing countries still rely heavily on wood fuel for heating and household purposes. While 40% of the global population (2.7 billion people) rely on traditional use of biomass as a source of energy in households (IEA 2010). Biomass commonly used in such as the three-stone fireplace or inefficient cookstove.

In rural Africa, trees are cut down forests for two purposes, burnt to make charcoal, wood fuel and land for farming. Charcoal traditionally made from tree species that yield dense, slow-burning charcoal (Girard 2002). However, the high demand for charcoal is leading to depletion of the species, posing a severe threat to deforestation as the population of Africa continue to grow, conversely, the need for new forms of energy sources increases. To date, wood fuel remains the primary source of energy in households, including micro-businesses involved in cooking foods.

Consequently, it is estimated 90 per cent of the entire population of Africa, uses fuelwood for cooking, and firewood and bushes, supply approximately 52 per cent of all energy sources (Agyei 1998). Other sources of energy, classified as non-traditional, are such as the burning of biomass, including sawdust. Sustainable uses of natural resources, especially forests, have proved to be challenging among others, weak policy framework in developing nations. In that, forests are burned down and release about 2.1 billion tons of carbon dioxide emissions into the atmosphere, of which 31% is from Africa (Pearson et al. 2017). It is difficult to say how much of forest burning, industry or motor vehicle contribute more given the latter is significantly small. The significant compounds emitted into the atmosphere include carbon dioxide (CO2), nitrogen dioxide (NO2) and other greenhouse gases (Sota et al. 2014). As the situation stands, high carbon emissions pose a considerable challenge, given that human activities contribute (Nyong et al. 2007). Besides wood burning as a contributor to pollution automobile traffic, industrial and mining are other sources of pollution in Africa. Mining activities, though, contribute to pollution on rural inhabitants. As earlier mentioned, global warming is increasing, and evidence from selected studies shows that prolonged droughts and reduced rainfalls have occurred in most parts of Africa over the last decades (Nhemachena et al. 2014). Moreover, debates on these topics are top of the list with civil society actors, environmental experts, and academia. Although concerted efforts towards developing environmental interventions are global, monitoring the progress remains paramount.

Climate is a unique good, as it contributes to livelihoods and is the basis for the existence of natural resources on the planet. However, the biggest threat to the coexistence of nature and humans are pollutions of greenhouse gases (Michaelowa 2007), contributing to global Warming (Schleussner et al. 2018). Naturally, a stable climate supports food production and wellbeing of the people. Thus, persistent drought in Africa is a very serious problematic (Katikiro and Macusi 2012). Studies on the climate change phenomenon in Africa show inhabitants, especially rural farmers, have been adapting to climate change by using traditional methods such as digging ditches to collect water (Kihila 2018). There are several interventions implemented towards reducing global greenhouse gases emissions and pollution to the environment (Brown et al. 2009), poverty alleviation, and climate change (Michaelowa 2007). These issues pose a serious problem to the wellbeing of people; in that, it is difficult to reduce high poverty levels if climate change continues to affect rainfalls.

Biomass remains the primary source of renewable energy, and the development of improved clean cooking stoves and small solar panels has penetrated the developing nations fast (Mamuye and Teshale 2018; Jurisoo and Lambe 2016). The global mission is to encourage the adoption of clean technologies that use renewable energy to help reduce pollution. Evidence shows that the adoption of cleaner energy for use in households is helping reduce high levels of poverty because of reduced cost (Basil et al. 2018). This paper refers clean technology, or cleantech, as an industry, a technology, and a solution that minimizes emissions of hazardous gases and uses renewable energy (Kachapulula-Mudenda et al. 2018; Alhola et al. 2014). The transition to adopting renewable energy-based clean stoves is slow in Africa because people, especially in rural areas, find it hard to change their traditional wood-based stoves.

Over the last few decades, the term “clean stove” interchangeably describes stove models that optimize fuel efficiency or designed to minimize hazardous emissions. Thus, their effectiveness in reducing indoor pollution that contributed high respiratory illnesses has been substantial (Rehfuess et al. 2014; Nyong et al. 2007). However, evidence from community health studies shows that using cleaner energy in households have significant health benefit because respiratory diseases from indoor pollution are low. There is still limited research showing how using improved clean stoves is impacting positively on both livelihoods and climate change, and how it has evolved in Africa. However, the research agenda has seen a significant growth in interest with 52% (*n* = 18) of the peer-reviewed articles reviewed here being published in the last six years (from 2003–2019). Despite this increase, however, this research identifies a research gap between interventions and society, primarily focusing on rural inhabitants in Africa has made limited progress in achieving impactful results.

This paper systematically reviews the literature to understand how research has evolved in tackling the challenges of pollution, environment, and climate change, and whether clean technologies as interventions are effective solutions in Africa. The following research questions guide the review: how useful are renewable-based techniques in reducing pollution, climate change, and environmental degradation; what are the positive impacts of clean technologies on the environment and livelihoods in Africa? The answers to this research question aim to contribute to the current shortage of studies on the effects of pollution on climate change and the people on the Africa continent. To improve understanding and knowledge of how the transition to using clean technologies is unfolding, including guide decision making to enhance climate intervention and action plans. This systematic review aims to understand the current focuses and area of research in Africa, on the effectiveness of clean technologies in reducing pollution, climate change and their impacts people’s livelihood. Very few clean technologies or correctly clean stove initiatives to date have demonstrated reduced climate change, and neither have concrete achieved disseminated. Consequently, a systematic review is likely to provide relevant cues on current research and direction taken to address critical problems of climate change and how this possible to guarantee the success of future initiatives and research.

## Methods

### Scope of the Systematic Literature Review (SLR)

This paper employs an SLR method to examine available research on the role of clean technologies in reducing pollution and environmental deterioration conducted in Africa between 2003 and 2019. The SLR is a literature review methodology commonly used to analyze the state of knowledge concerning a particular topic (Petticrew and Roberts 2011). SLR is widely in use on issues of climate change applied to analyze and interpret the status of experience and aid in identifying and reshaping the directions for further research efforts (Williams et al., 2018). In principle, cleaner technologies encompass a variety of technological designs, ranging from domestic solar panels installation, biogas stoves and user-built stoves, all made of locally in Africa. The investigation started with the choice of a suitable database. Both Webs of Science and Scopus are well known as comprehensive databases covering well the research done in the global academic community. The first trials indicated that in terms of climate change, clean technology, and Africa, the two databases produced remarkably similar results. Therefore, we chose to focus only on Web of Science as adding a second data source did not seem to add much value. Next, we decided the time frame for investigation and the keywords used for the search. Our literature review focused on a period of 20 years, ranging from 2003 to March 2019. This period was appropriate in producing a substantial volume of papers to enable a holistic screening. By noting that pollution comes because of human activities, including several significant factors operating at all levels in society, we set up this review to encompass four types of studies:

- Qualitative studies, as intervention or single studies.
- Quantitative linked to health studies.
- Case studies drawn from multiple sources of information provide an assessment of a context project, program, or policy strategies for continental Africa.

**Fig. 1:**
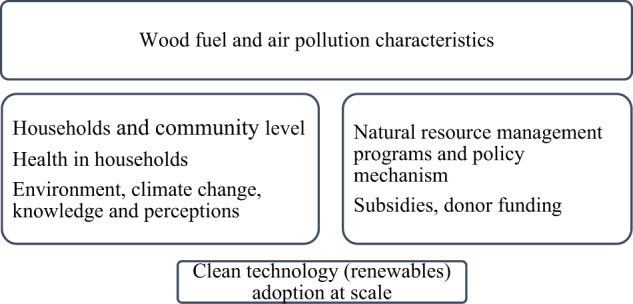
The framework illustrates the impact of air pollution and intervention ecosystem

To undertaking a rigorous review, we began by restructuring it by developing a comprehensive framework of essential elements interfacing with the impact of pollution and interventions of clean technologies (Fig. 1). This framework encompasses six factors.

The framework highlights the impact of wood fuel and atmospheric and indoor air pollution characteristics trickling down to renewable energy-based solutions. Also, aspects of households and settings embrace knowledge and perceptions that primarily operate at the household and community level. Enabling or limiting factors affecting short term clean technology adoption may differ from those involving longer-term sustained use. Also, scaling up renewable energy use may occur equally or unequally across the population varying by socioeconomic status in either urban-rural location, and it is can as well influenced by gender-related factors.

The review involved an in-depth analysis in producing a comprehensive overview, as outlined in the next section. Furthermore, identifying eligible studies, titles and abstracts were screened by authors, with independent random checks of included and excluded abstracts. Authors independently screened the full text of articles for initial consideration to ensure rigorous review.

### Data Selection Process

To ensure adherence to academic procedures for conducting a systematic review, we followed the guide from the book by Petticrew and Roberts (2011). A research protocol developed, as shown in Table 1. The protocol reported the inclusion and exclusion criteria for our literature review as proposed by (Williams et al. 2018).

**Table 1:** Criteria for literature selection

Search protocol	Inclusion criteria	Exclusion criteria
Initial database and literature search	English literature	Non-English literature
	Quantitative and Qualitative	
	Peer review articles	
	Single studies	
	Studies in Africa and developing countries	
Review of title and abstract	Clean technologies	They are explicitly focusing on the ocean, aquatic, and atmospheric. Ecosystems
	Pollution reduction	
	Climate change mitigation and adaptation	
	Technology adoption	
	Geographically global	
Full paper	Original studies	Project reports
	Pollution, climate change mitigation and clean technology	Policy documents
	Renewable energy, environment, and natural resources studies	Non-peer review

In considering the eligibility for inclusion/exclusion of the studies, we focused only on articles that had key words of climate change and clean technology effect related to Africa. We adopted a comprehensive search strategy comprising a systematic search in peer-reviewed databases. Initially, we performed trials on google scholar, Web of Science and EBSCO. However, we ended up selecting a single database, the Web of Science, because of its extensive search results. In the search process, we refined the search with the following keywords:

1. [Clean technolog* effectiveness OR Cleantech effectiveness], [clean technolog* transfer], [Climate* AND Africa].

2. (clean technolog* effectiveness) OR TOPIC: (cleantech* effectiveness) AND TOPIC: (climate*) AND TOPIC: (Africa*).

**Fig. 2:**
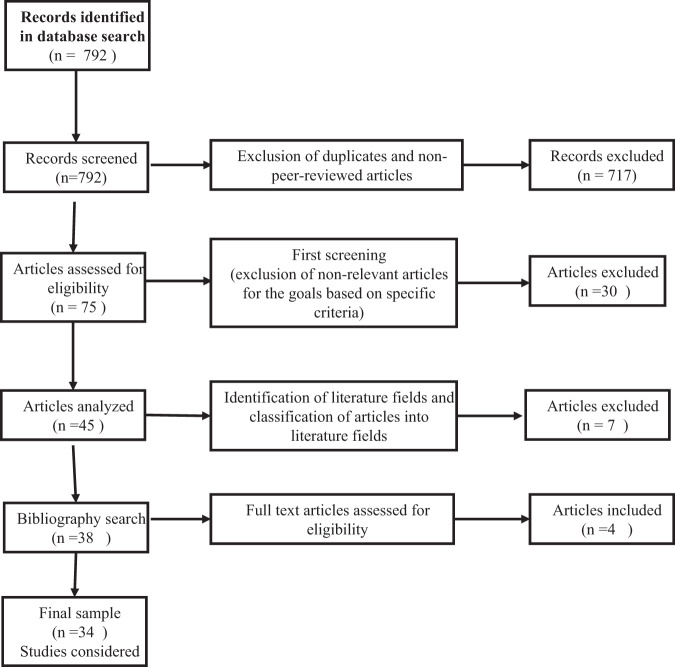
Summary diagram of the literature selection process

The initial search produced a total of 792 articles from the Web of Science database constituting peer-review articles and other publications (working papers, conference papers, project documents, and published theses). The first phase of screening involved removing non-peer-reviewed articles from 792 articles; about 717 articles were non-peer-reviewed. As a result, 75 articles were chosen for eligibility review and assessed in terms of their relevance to the topic. In this phase, 30 articles excluded. The remaining 45 articles went through a rigorous evaluation process to ensure that they matched with the criteria regarding the issues of pollution, climate change, and clean technology studies in Africa with a focus on the impact of mitigation factors. The result after this lengthy process of finally reaching the 34 peer-reviewed articles is shown (Fig. 2).

**Fig. 3:**
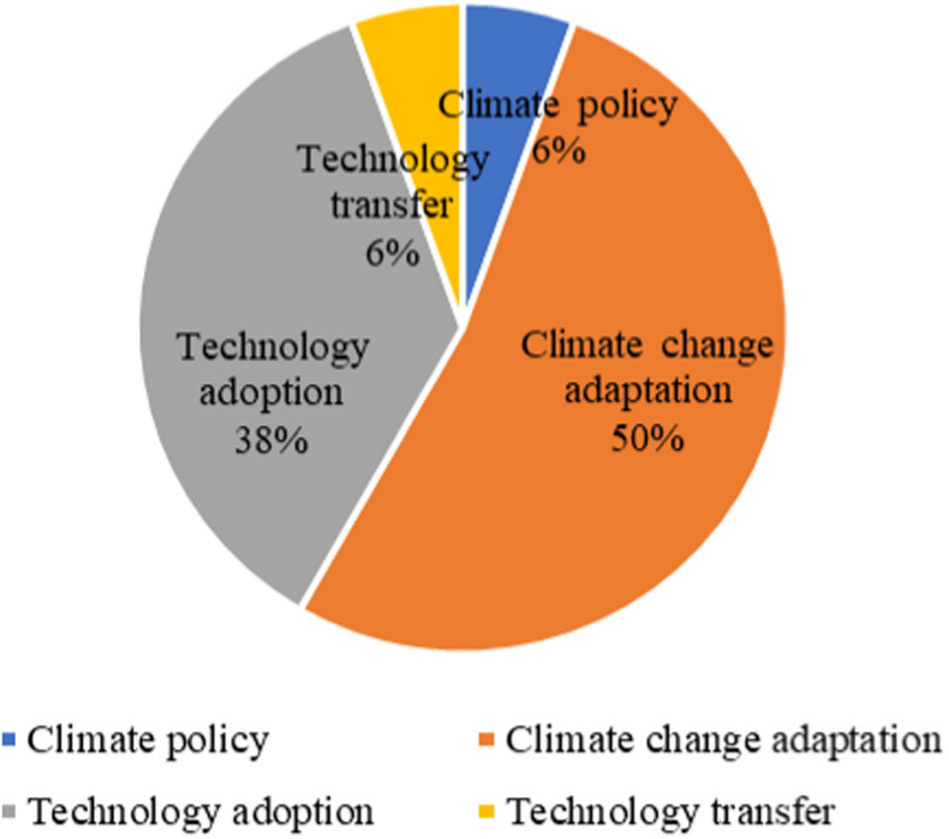
Share of research domain in Africa (2003–2019)

The data extraction exercise for included studies was conducted by the author using the standard excel sheet. Even though included studies were peer review articles from scientific journals, further review on international project reports and policy documents proved relevant. The studies assessed ensured consistency by validating the information source that matched country details and authors affiliation. This review includes the volumes of studies concerned with the research area of the 34 studies reviewed, as shown in Fig. 3. Notably, the number of studies of research topics included five sets; Climate policy (*n* = 2), Technology adoption (*n* = 13), Climate change adaptation (*n* = 17) and Technology transfer (*n* = 2) were a division of contributions. A concise synthesis of the findings from each research domain provided to show the weight of contributions.

Next, we present the share in percentages of each research domain shown in a pie as percentages (Fig. 3), and provide evidence synthesis of the assessments of the reviewed articles as follows:

#### Climate policy

A significant number of studies reviewed were concerned with issues of climate change. Of the 34 studies considered, only 6% assessed the pros and cons of climate change policies on Africa. The reviews try to identify the gaps and weaknesses in the policy framework by elaborating on the failure of climate change mitigation in many countries in Africa. Further, it relates to issues of implementation and resource allocation of the global agreements on climate change.

#### Climate change adaptation

This category the highest volume of studies 50% (*n* = 16) from the total of 34 articles reviewed which indicates the concentration of research on the topic across Africa. We synthesis studies by analyzing the critical review from Nath and Behera (2011) which summarises that poorer countries and communities could suffer the most because of their geographical locations, low income, weak governance, and inadequate institutional capacity. Notably, African countries rely heavily on climate-sensitive sectors, such as agriculture but the current situation require a new adaptability approach. Nath and Beth (2011), find that the developed and developing countries adaptation strategies, work better when there is a synergy between climate change initiatives and socioeconomic policies. Similarly, Katikiro and Macusi (2012), stresses on the need to strengthen local to regional policies to address adaptation to climate change on the case of empowering fishing communities to improve livelihood. The developing countries though, are having lower rates of emissions than developed nations.

#### Technology adoption

The findings for this domain derived from the Clean Development Mechanism (CDM) which is the primary channel for cleantech promotions. From the reviewed literature, several initiatives for reducing air pollution and the development of renewable energy-based technologies attracts many researchers (Karanja and Gasparatos 2019; Jurisoo and Lambe 2016; Olawuyi 2017), whose studies involved aspects of people’s transition from using traditional wood fuel to solar and clean cooking stoves. However, upon synthesizing these studies, it is clear that the adoption of clean cooking options is expanding, but it is notably slow throughout Africa. Of the 34 studies, 38% (*n* = 13) focused on technological adoption. Africa has abundant sunshine and based on this potential natural energy resource can be tapped into consumable energy through the solar panel that can suit most households, especially in remote rural areas. However, large-scale solar energy plants are to replace traditional hydroelectricity, which has become susceptible to climate change due to long spells of drought experienced across Africa. Despite this, a study by Maji (2019) argues that renewable energy has a significant indirect impact on CO2 emissions which implies that the increases in the use of renewable energy reduce CO2 emissions in Africa.

#### Technology transfer

Technology transfer provides several potential benefits. As noted from reviewed studies, 6% (*n* = 2) were concerned with technology transfer under the north to south models. In a study by Dechezlepretre et al. (2014) find that a broad range of clean technologies, including renewable energy technologies and other carbon capture and storage technologies made in developed countries. Similarly, the findings draw from among other studies like Olawuyi (2017) also highlighted in, Koefoed and Buckley (2007) both confirm that technology transfer as predominately flowing from north to south. However, access to technological solutions is not readily available in many African countries. Another significant finding is on the policy structures across regions of Africa, where a policy in one part can affect technological innovation in another section. The socioeconomic dimension involving income levels, literacy, and household assets, is critical to enabling technological adoption. Besides the study by Popp (2011), emphasized that international trade and foreign investments help catalyze the transfer of cleaner technologies to developing countries, including Africa.

## Results

### Description of the selected studies

**Fig. 4:**
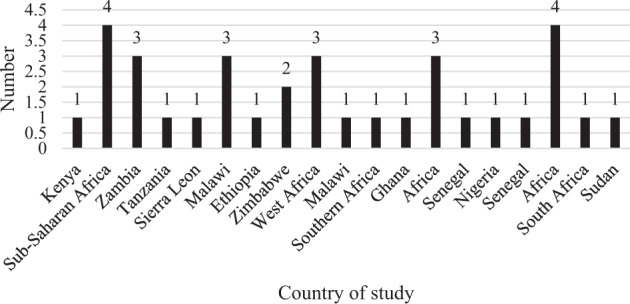
Number of scientifical articles conducted in specific countries

The search identified 792 articles from which, 34 studies met the criteria. Selected articles included qualitative, quantitative, and case studies spreading across Africa. Subsequently, South Africa (*n* = 10), West Africa (*n* = 9), Kenya (*n* = 5), Nigeria (*n* = 2) and Malawi (*n* = 4) contributed many studies, see Fig. 4. Of the 34 studies, 30 were undertaken in single African countries areas, whereas 4 covered the whole continent. Table 1, shows basic study characteristics: 35 studies were concerned with adoption and 13 studies with sustained use, whereas nine studies assessed elements of both adoption and sustained use. Detailed study characteristics appear in Table 2, which show thematic studies determined by references comprising research domain, country or region and date of publication.

**Table 2:** Basic study characteristics

Reference	Research description	Study country/ region
A. Climate change adaptation		
1. Schleussner et al. 2018	Climate Change	Sub-Saharan Africa
2. Kihila 2018	Climate Change	Tanzania
3. Gautier et al. 2016		West Africa
4. Mabhaudhi et al. 2017		Southern Africa
5. Frimpong et al. 2015		Ghana
6. Shackleton et al. 2015		Africa
7. Nhemachena et al. 2014		Zimbabwe
8. Olsson et al. 2016	Pollution	West Africa
9. Katikiro and Macusi 2012	Agriculture (fisheries)	West Africa
10. Brown D., 2011	Renewable energy, climate	Malawi
11. Brown et al. 2009	Agriculture (food security)	West Africa
12. Nyong et al. 2007		Kenya
13. Stephenne N., & Lambin E.F., 2004.		Africa

B. Clean technology adoption		
1. Karanja and Gasparatos 2019	Renewable energy/pollution	Kenya
2. Kachapulula-Mudenda et al. 2018		Zambia
3. Chirambo 2018		Malawi
4. Chirambo 2018		Malawi
5. Jürisoo and Lambe 2016		Zambia
6. Rehfuess et al. 2014		Sub-Saharan Africa
7. Mohammed Y.S. et al., 2014		Nigeria
8. Cabral F.J., 2012		Senegal
9. Koefoed and Buckley 2007		South Africa

C. Technology transfer		
1. Sam M & Simwela A., 2018	Focuses on the north-south technology transfer, air pollution implications	Sierra Leon

D. Climate Policy		
1. Gueye, C. et al., 2015	Climate Change	Senegal
2. Michaelowa and Michaelowa 2007		Africa

Many studies were concerned with clean stoves developed by local artisans, and seven studies contributed findings for more standardized stove production. The qualitative studies were part of climate change interventions and the eligibility of the studies determined through keywords from the titles and abstracts. The process also included random checks of included and excluded abstracts. The reason for selecting articles using several methodologies was to ensure the holistic inclusion of all relevant studies. The qualitative studies comprised interviews, focus groups, community-based education, and ethnographic studies, while the quantitative studies included cross-sectional surveys and socioeconomic analyses. The case studies’ data collection methods varied, including, for example, surveys, interviews, and focus groups. There was mainly one study per country, but a more significant number of the article were concerned with education for the whole continent of Africa (Fig. 4). We ensured that eligibility for the inclusion of the studies is related directly or indirectly to topics within African climate change subjects. Most of the studies provide empirical and qualitative information on critical factors dominating climate change agenda. Our analysis of research contributions shows that research on climate change is not solely governed by local universities but through collaboration with international environmental institutions.

The studies on climate change did not usually focus on a single country but covered a broader region or the whole continent. Among these studies, four focused on Sub-Saharan Africa and Africa consecutively. In terms of specific country studies, the research question or theme of the study is representative of the problem matter dominating the continent. The study characteristics show a list of all 34 articles, Table 2, offers primary study characteristic in terms of study focus, and research domains as [*A, B, C and D*], with some of the authors. Significantly, studies in technology transfer predominately conducted by the Western higher institution of learning. Consequently, 17 items (50%) of articles were concerned with climate change adaptation, as the primary research focus area and 13 related to adoption strategies of clean technologies. Most studies were concerned with aspects relating to how communities are and have been adapting in developing nations, especially Africa, and 13 studies contributed findings on the dynamics of clean technology adoption focusing on clean cooking stoves.

The reviewed articles included qualitative studies comprised interviews, focus groups, and ethnographic studies. Descriptions of the theoretical approach to analysis and of strategies employed to increase the validity of findings, as well as limited distinctions between conclusions emerge from the research and subjective author interpretations, were common problems. The analysis of the articles involved objectives and findings. Therefore, the studies were a mix of qualitative, quantitative, and case studies on Africa. We noted that many quantitative studies comprised controlled cross-sectional surveys and economic analyses. The main areas of weakness were inferior sampling methods and relatively simple descriptive investigations. However, the case studies varied dramatically in terms of the combination of direct empirical findings in the case of cross-sectional surveys, focus groups, interviews reference to publicly available statistics.

The number of contributions in scientific journals in Environmental Sciences was significant, and the review included sixteen such articles altogether. In considering the available evidence, a large volume of research is predominantly in renewable energy as mitigation against excessive pollution in Africa. Research on biomass development as a renewable energy source has been considered essential for the sake of human health, protection of the environment, and climate change abatement. African research on climate change is confirmed to scale up a study in climate change adaptability as a significant intervention. The increase in the volume of publications from 2014 to 2018, concentrating on topics climate change adaption and technology transfer.

**Fig. 5:**
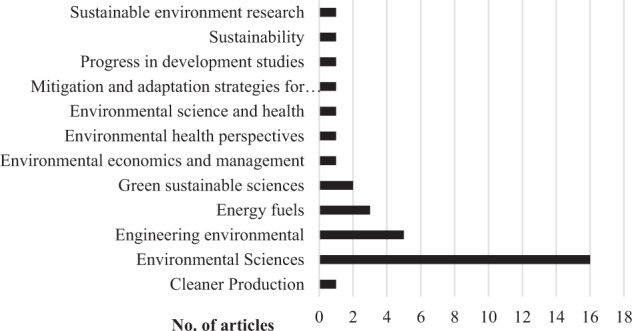
Number of articles reviewed in Scientific Journals

As for the composition of the scientific journals, the final sample included articles from the following, see (Fig. 5), generated from the Web of Science categories: environmental sciences, public environmental, occupational health, green sustainable science technology, energy fuels, agronomy, water resources, environmental studies, economics, and atmospheric meteorology sciences. Articles from the following journals: law or political science or toxicology. Most of the studies were concerned with issues in environmental sciences with a volume of 50% (*n* = 16), the highest number of articles considered for review.

### Current Knowledge Base

There is overwhelming evidence that rising temperatures and global greenhouse gas (GHG) emissions linked to climate change phenomenon (Intergovernmental Panel on Climate Change (IPCC) 2018). Besides, anthropological literature on climate change has equally supported the assertion based on the studies of societal and cultural perspectives of the human populations over a long period (Crate & Nuttall, 2016). The same body of literature brings to light that human societies have evolved over centuries through adapting to severe climatic conditions, which confirms that the climate change phenomenon has existed over time. Similarly, this supports (Nyong et al. 2007) study of the African Sahel that has been characterized by recurring droughts. However, their finding is that despite the severe climate conditions, the indigenous people of the Sahel adapted well to harsh climate conditions for more than 100 years.

Adaptation methods are those strategies that enable the individual or community to cope with or adjust to the impacts of climate in their local areas (Nyong et al. 2007). Indigenous knowledge has not been adequately applied in most climate mitigation strategies because renewable technology is attracting more attention. Adaptation to climate change occurs when people perceive changes in climate phenomena, such as prolonged drought (Nhemachena et al. 2014). A large volume of literature reviewed on climate change adaptation cites policy mismatch, as noted in Brown et al. (2009), emphasizing the need for planned adaptation measures across Africa. Emphasis on information dissemination for actions towards reducing carbon emissions across rural and urban regions is critical. The world population rely on everyday energy requirements on fuels. While most of the African people rely on wood fuels as earlier discussed. Notably, the transition rate from using wood fuel to renewable fuels is slower in low-income countries than developed nations (Rehfuess et al. 2014). However, far too often, the construct of a particular policy determines the success of meeting specific goals in climate change interventions.

As a means for structuring the review, a comprehensive framework of studies and research domains completed in various African countries shown in Table 2. The current plan in most African countries on climate change focuses on the adoption of clean technology and renewable energy. There is also a considerable involvement of private, cleantech companies that sell solar panels and clean cookstoves. The clean technology adoption programs involving clean cookstoves developed on the basis that their functionality has a positive impact on health and the environment (Mamuye and Teshale 2018). Besides, they contribute to social and economic benefits through their adoption (Akram et al. 2013). Despite this, Africa has a low rate of technological adoption, and most of the population cannot access electricity, particularly when compared to other continents (Olawuyi 2017) and (Kachapulula-Mudenda et al. 2018). That is especially true for the type of technology that contributes to pollution reduction.

Even though there are significant improvements in electricity distribution, of up to 43% in Africa, the slow pace of inclusivity in rural areas is an issue of concern. Given the ever-increasing human population experienced across the African continent (Dahunsi et al. 2020). Several international cleantech programs, such as the Clean Development Mechanism (CDM), focus primarily on the adoption of technology (Michaelowa 2007). To reach the remote rural regions of Africa, CDM implements projects in these areas on a large scale. The cost of climate-friendly technologies has been high for the past decades but is now subsidized or tax-free for developing countries (Dechezlepretre et al. 2014). Currently, the direction of research on renewable energy solutions focuses primarily on adoption biogas cookstoves (Karanja and Gasparatos 2019; Mamuye and Teshale 2018; Kachapulula-Mudenda et al. 2018) and affirms that fresh cooking can help to achieve greater fuel energy efficiency while improving people’s health.

## Discussion

Over the last decade, the rise in assessing environmental impacts and research in Africa has begun to focus on smallholder livelihoods and communities. Notably, there is a steady rise in annual studies, as evident since 2000. In the last two decades, there has been growing interest in research concerning climate change and related impacts on livelihood and economies. Drawing on the 34 studies with qualitative, quantitative, and case-study designs, in the present systematic review, we identified 16 distinct factors contributing to Africa’s adaptabilities methods to changing climate and depletion of forests. An integration between factors primarily acting at the community level and factors operating mostly at the program/ societal level is critical if plans are to reach their intended populations and be successful at scale and over extended periods.

In several African countries, the rate of deforestation exceeds the global annual average of 0.8 per cent (Agyei 1998). Though deforestation in other parts of the world mainly caused by commercial logging or cattle ranching, in Africa, the leading cause is associated with peoples livelihood activities. Consequently, an estimated 90 per cent of the continent’s population uses wood fuel as a source of energy domestic purposes (Girard 2002). Traditionally, wood fuel refers to as fuel energy such as firewood, charcoal, and pellets, among others. Several studies reviewed, among others (Girard 2002; Karanja and Gasparatos 2019; Agyei 1998. Basil et al. 2018) point out the traditional use of wood fuel, including burning and clearing of land contributes to deforestation and pollution. While Agyei (1998) argues that one critical example concerning communal living and land tenure systems in Africa, is that it is challenging to allocate incentives to individual investment the land. However, traditional and communal land in Africa remains a challenge to external initiated sustainability interventions because conventional fabrics control there.

The consistency of the findings offers some guide to importance, of which this review fulfils. Though the analysis may have been optimal because of missing evidence, it does not mean that element is unimportant. For example, few studies report on the perception of clean technologies in African communities. Mostly they reflect a lack of the policy construct to address critical issues in this field. Notably, attempts to identify the fundamental problems can, in this case, rely mainly on the judgment. It appears that several of the climate intervention elements identified, spread across household and community themes primarily, socioeconomic aspects, perceived benefits, and traditional and culture. However, the most critical determinate in enabling the adoption of cleantech products like clean cooking, was to make sure that they met formal cooking requirements, significantly when a household initially persuaded to acquire it. Notably, principles of diffusion of innovation theory entail that perceived overall clean cooking stove benefits exceed traditional fuelwood cooking practices. The adoption of stoves to a broader community requires effective service can set out benefits to be health, fuel energy, and time savings or preserved forests (Rehfuess et al. 2014; Jurisoo and Lambe 2016; Karanja and Gasparatos 2019). However, considering that traditional cooking is proving unsustainable with an adverse effect on forests, health and the environment, these aspects differ across social settings and social strata.

### Intervention Effectiveness

Technological development is still weak in many African countries, which makes them recipients of new technologies (Olawuyi 2017). As noted in the literature on new climate technologies, the phenomenon of technology transfer from developed to developing countries is still active, which confirms the application of the North-South model (Popp 2011). The most significant shift is that new technologies are now coming from Asian countries. For example, the development of renewable energy, biogas existed in China for centuries, currently being promoted in Africa (Karanja and Gasparatos 2019). Significantly, about half of the studies reviewed tackling issues of climate change adaptation while a small number focused on technology adoption. It clearly shows that climate change adaptation matters are of considerable importance in research on African climate change. Evidence shows that the technological gap between developing and developed countries is wide (Olawuyi 2017). However, some studies find that African collaboration with international promoters of clean cooking stoves is proving useful in terms of high adoption in East Africa (Mamuye and Teshale 2018; Karanja and Gasparatos 2019). It is also helping to reduce carbon emissions, and consequently, aiding environmental sustainability, especially in deforestation.

### Methodological Strength and Limitations

This review provides a comprehensive status of current research and the existing knowledge gaps regarding the impact of clean technology on climate change. The report identifies two areas of research. The first one covered a study that tries to promote the use of clean technology and analyses on knowledge gap in societies. Studies that focused on the rural area had better findings regarding the lack of capacity-building instruments in using clean technologies, such as the clean cooking stoves and biogas supported by Karanja and Gasparatos (2019). The authors admit that research does not focus on the impact of technology and what it has achieved in addressing climate problems rather than emphasizing technological adoption.

### Clean Technology Adoption Versus Wood Fuel, Versus Deforestation

Traditional and inefficient cooking practices can potentially have severe environmental impacts, mainly linked to ecosystem degradation and greenhouse gas (GHG)emissions (Karanja and Gasparatos 2019). Thus, interventions to reduce the negative effect have engaged in promoting the adoption and sustained use of clean-fuel and energy technologies. Clean cooking stoves belong to the group of clean technology ‘cleantech’, comprise products and services that optimize on the use natural resources and offer a more decent alternative to traditional products and services (Alhola et al. 2014). Notably, promoting clean cooking technologies can best constitute substituting from using traditional firewood to efficient biomass stoves or fuels that help curb negative sustainable impacts. Our review did not specifically explore deeply into how communities perceive the transforming from using traditional wood fuel to renewable energy because of their limited studies. Indeed, as previously reported in the present review, clean cooking stove adoption does not imply that households abandon their traditional use of wood fuel but reduce cutting down trees for fuel. The analysis also shows that factors influencing clean technology adoption vary from country to country, depending on the availability and proximity of tree areas.

For this result, the review shows a specific gap in research areas that are trying how to understand how clean technology is benefiting communities in terms of an intervention to the effects of climate change developing countries. It is essential to strengthening our understanding of the factors are most valuable when implementing clean technologies in the global south towards the negative impact on the environment. The adoption of technologies is critical (Popp 2011; Karanja and Gasparatos 2019; Dechezlepretre et al. 2014). Therefore, this review proposes a need for more robust research in identifying innovative measures that sustain capabilities to adapt to adverse causes of climate change, particularly GHG emissions. In the long term, it will require broadening the scope of both quantitative and qualitative scientific approaches. For example, Africa is a diverse continent and pretty challenging to meet a specific goal related to implementing any such interventions.

Nonetheless, qualitative methods can make a significant contribution to ensure an understanding of the uptake process, especially by capturing stakeholder perspectives that include those of beneficiaries, rural communities, government, and industry. Prospective evaluations of international clean technology programs that incorporate the findings of this review include a focus on the clean cooking stove, which can contribute to reducing the depletion of forests. Future research agenda should take up a holistic approach to develop tools and framework for scaling forest sustainability intervention and renewable energy technologies.

We note that from most studies concerned with pollution, also tackle the impacts of using fuelwood which mainly advocated through donor agencies. To eliminate the open-access area for firewood, it is relevant to regulations that promote the market of clean products which will help decrease deforestation. Africa endowed with abundant forests; however, issues around policy and traditional systems of land ownership make sustainability initiatives challenging. Other significant measures to deforestation are through the agroforestry market, where tree farming is involved, levies on transporting fuelwood, and setting up of forest reserves restricting the areas access to cut trees for fuel or other purposes. While these strategies are right, however, in the traditional African societies are challenging for such measures to work. Notably, in countries where there is a mismatch on policy versus standard rules because the government may have little control to rural regions were large areas may under control of traditional leaders. Even for strategies that require a nationwide collection of fees or enforcing rules on harvesting fuelwood are nearly impossible. African countries do not have the capabilities in key institutions that can necessitate the operation of such a framework.

## Conclusions

This SLR provided a comprehensive outlook of the current state of research in carbon emissions and uses of clean technologies, and their impact towards improving livelihoods in Africa. The findings suggest increased fuelwood consumption and the increasing population are contributing to the depletion of the forests. At the same time, clean technology specifically clean stoves adoption is low, and that so far there is no substantial evidence to show its effectiveness towards reducing climate change. In this systematic literature review (SLR), we identified five dominant factors that contribute to intervention strategies applied for air pollution, clean technology, and climate variability in Africa. The selected articles show that 50% of the studies belonged to topics on climate change adaptation, followed by 38% on technology adoption. Specifically, energy consumption in Africa is still low, albeit fuelwood is still leading source energy for the majority of the population. Energy consumption drives economic growth while at the same time, support livelihood. However, the production processes impact negatively on the environment and instead contribute to outdoor pollution. Therefore, adding the adoption of clean, renewable energy options and innovative natural resource sustainability measures to the mitigation mix can contribute to the reduction of pollution and the use of firewood. In turn, it would help increase economic productivity while reducing environmental degradation and pollution in Africa. The findings suggest providing the basis for the development of a policy tool that integrates all research domains reviewed, which would encapsulate in planning interventions, implementation, and evaluation stages of policies and programs promoting specific renewable energies in particular settings. In far, as environmental interventions are concerned, an inclusive society, clean technology industry and initiatives sustainability in the natural resources considered.

For future research, we propose two steps: (1) Based on recent statistics (World Resources Institute), show detailed and specific findings that Africa has the fastest-growing population; therefore, we propose a research agenda that will integrate demographic elements. As stated, the annual population growth rate is approximately 3 per cent, which poses a severe problem to limited natural resources, given that it is twice the world growth rate of 1.7 per cent. Besides, life expectancy has increased, while the infant mortality rate decreases. Based on this information, the population in Africa expects to double over the next 20 years. Therefore, demand for wood fuel energy will increase, and research agenda should focus on multi-level sustainability approach of clean technology versus pollution on one hand forests on the other. (2) To broaden our understanding on the factors that are most important for securing the adoption and sustained use of clean stoves, including maintenance and replacement, to be drawn from a combination of quantitative and qualitative scientific approaches. Overall, we recommend more qualitative research to significantly contribute towards ensuring deeper understanding by capturing perspectives in communities, including those of beneficiaries, government, and industry. We conclude by suggesting developing prospective evaluations of programs to include a focus on the complexities and controversial topics on stove subsidies that will significant in long term reduction on the impact of pollution.

## References

[CR2] Agyei Y (1998) Deforestation in Sub-Saharan Africa. *African technology forum* 8(1)

[CR3] Akram W, Irfan M, Iqbal S, Waqar I (2013). The perception about the biogas technology adoption: a case study of district Faisalabad (Punjab, Pakistan). Middle-East J Sci Res.

[CR4] Alhola K, Nissinen A, Seppälä J (2014). Promoting cleatech and public procurement and investments in Finland.. Glob Public Procurement Theories Pract.

[CR5] Basil AS, Martinus K, Tchakert P, Wills R (2018). Disruptive innovations and decentralized renewable energy systems in Africa: a socio-technical review. Energy Res Soc Sci.

[CR6] Brown D (2011). Making the linkages between climate change adaptation and spatial planning in Malawi. Environ Sci Policy.

[CR7] Brown ME, Hintermann B, Higgins N (2009). Markets, Climate Change, and food security in West Africa.. Environ Sci and Technol.

[CR8] Cabral FJ (2012). The impact of rainfall fluctuations on regional disparities in poverty (Senegal) [L’impact des aléas pluviométriques sur les disparités régionales de pauvreté au Sénégal]. Rev d’Economie du Dév.

[CR9] Chirambo D (2018). Towards the achievement of SDG 7 in sub-Saharan Africa: Creating synergies between Power Africa, Sustainable Energy for All and climate finance in-order to achieve universal energy access before 2030. Renew Sustain Energy Rev.

[CR10] Crate SA, Nuttall M (2016). Anthropology and Climate Change: From Encounters to Actions.

[CR11] Dahunsi SO, Fagbiele OO, Yusuf EO (2020). Bioenergy technologies adoption in Africa: a review of past and current status.. J cleaner prod.

[CR12] Dechezlepretre A, Martin R, Mohnen M (2014) Knowledge spillovers from clean and dirty technologies. *Grantham Research Climate change and the Environment Working paper No*. 135

[CR13] Frimpong K, Van Etten E, Oosthuzien J, Fannam VN (2015). Review of climate change adaptation and social protection policies of Ghana: The extent of reducing impacts of climate change and heat stress vulnerability of smallholder farmers. Int J Soc Ecol Sustain Dev.

[CR14] Gautier D, Denis D, Locatelli B (2016). Impacts of drought and responses of rural populations in West Africa: a systematic review. Wiley Interdiscip Rev: Clim Change.

[CR15] Girard P (2002). Charcoal production and use in Africa: what future?.

[CR16] Gueye C, Fall AS, Tall SM (2015). Dakar, Touba and the Senegalese cities network produced by climate change. Curr Opin Environ Sustainability.

[CR1] IEA (2010). World energy outlook 2010, energy poverty. How to make modern energy access universal?.

[CR17] Intergovernmental Panel on Climate Change (IPCC) (2018) Global Warming of 1.5 degrees celsius. IPCC special report, France/UK

[CR18] Jurisoo M, Lambe F (2016) *The journey to clean cooking: Insights from Kenya and Zambia*. Stockholm: Working paper, Stockholm Environmental Institute

[CR19] Kachapulula-Mudenda P, Makashini L, Malama A, Abanda H (2018). Review of renewable energy technologies in zambian households: capacities and barriers affecting successful deployment. Rev Build.

[CR20] Karanja A, Gasparatos A (2019). Adoption and impacts of clean bioenergy cookstoves in Kenya. Renew Sustain Energy Rev.

[CR21] Katikiro RE, Macusi ED (2012). Impacts of climate change on West African fisheries and its implications on food production. J Environ Sci Manag.

[CR22] Kihila JM (2018). Indigenous coping and adaptation strategies to climate change of local communities in Tanzania: a review. Clim Dev.

[CR23] Koefoed M, Buckley C (2007). Clean technology transfer: a study from South Africa metal finishing industry, 200-2005. J Clean Prod.

[CR24] Mabhaudhi T, Chimonyo VGP, Modi AT (2017). Status of underutilised crops in South Africa: Opportunities for developing research capacity.. Sustainability (Switzerland).

[CR25] Maji IK (2019). Impact of clean energy and inclusive development on CO2 emissions in sub-Saharan Africa.. Cleaner Production.

[CR26] Mamuye F, Teshale W (2018). Emission and fuel use performance of two improved stoves and determinates of their adoption in Dodola southeastern Ethiopia. Sustain Environ Res.

[CR27] Michaelowa AA (2007). Climate or development: is ODA diverted from its original purpose?. Clim Change.

[CR28] Mohammed YS, Mustafa MW, Bashir N, Ogundola MA, Umar U (2014). Sustainable potential of bioenergy resources for distributed power generation development in Nigeria. Renew Sustain Energy Rev.

[CR29] Nath PK, Behera B (2011). A critical review of impact of and adaptation to climate change in developed and developing economies. Environ Dev Sustainability.

[CR30] Nhemachena C, Mano R, Mudombi S, Muwanigwa V (2014). Perceptions on climate change and its impact on livelihoods in Hwange district Zimbabwe.. J Disaster Risk Stud.

[CR31] Nyong A, Adesina F, Elasha BO (2007). The value of indigenous knowledge in climate change mitigation and adaptation strategies in the African Sahel. Mitigation Adaptation Strategy Glob Change.

[CR32] Olawuyi DS (2017). From technology transfer to technology absorption: addressing climate technology gaps in Africa. J Energy Nat Resour Law.

[CR33] Olsson J, Arheimer B, Borris M, Donnelly C, Foster K, Nikulin G, Persson M, Perttu A-M, Uvo CB, Viklander M, Yang W (2016). Hydrological climate change impact assessment at small and large scales: Key messages from recent progress in Sweden. Climate.

[CR34] Pearson TR, Brown S, Sidman G (2017) Greenhouse gas emissions from tropical forest degradation: an underestimated source. *Carbon Balance and Management*. *12**3* 10.1186/s13021-017-0072-2 PMC5309188 28413847

[CR35] Petticrew M, Roberts H (2011). Systematic Reviews in the Social Sciences.

[CR36] Popp, D (2011) International technology transfer, climate change, and the clean development mechanism. Rev Environ Econ Policy 5:131–152

[CR37] Rehfuess EA, Puzzolo E, Stanistreet D, Pope D, Nigel BG (2014). Enablers and barriers to large-scale uptake of improved solid fuel stoves: a systematic review. Environ Health Perspect.

[CR38] Sam M, Simwela A (2018). Availability, Accessibility and the Road Map for Clean, Affordable, Effective and Efficient Energy for Sierra Leone; A six years Analysis from 2006 to 2011. Int J Sci Res Publ.

[CR39] Schleussner C-F (2018). 1.5°C hotspots: climate hazards, vulnerabilities, and impacts. Annu Rev Environ Resour.

[CR40] Shackleton S, Ziervogel G, Sallu S, Gill T, Tschakert P (2015). Why is socially-just climate change adaptation in sub-Saharan Africa so challenging? A review of barriers identified from empirical cases. Wiley Interdiscip Rev: Clim Change.

[CR41] Sota CD, Lumbreras J, Mazorra J, Narros A (2014). Effectiveness of improved cookstoves to reduce indoor air pollution in developing countries. the case of the cassamance Natural Subregion, Western Afric. J Geosci Environ Prot.

[CR42] Stephenne N, Lambin EF (2004). Scenarios of land-use change in Sudano-sahelian countries of Africa to better understand driving forces. GeoJournal.

[CR43] Williams PA, Crespo O, Abu M, Simpson NP (2018). A systematic review of how vulnerability of smallholder agricultural systems of changing climate is assessed in Africa. Environ res lett.

